# Perfluoroalkyl substances (PFASs) are substrates of the renal human organic anion transporter 4 (OAT4)

**DOI:** 10.1007/s00204-022-03428-6

**Published:** 2022-11-27

**Authors:** Jochem Louisse, Luca Dellafiora, Jeroen J. M. W. van den Heuvel, Deborah Rijkers, Liz Leenders, Jean-Lou C. M. Dorne, Ans Punt, Frans G. M. Russel, Jan B. Koenderink

**Affiliations:** 1 Wageningen Food Safety Research, Wageningen University and Research, Wageningen, The Netherlands; 2 Department of Food and Drug, University of Parma, 43124 Parma, Italy; 3 Department of Pharmacology and Toxicology, Radboud University Medical Center, Radboud Institute for Molecular Life Sciences (RIMLS), Nijmegen, The Netherlands; 4 Methodological and Scientific Support Unit, European Food Safety Authority, Via Carlo Magno 1A, 43124 Parma, Italy

**Keywords:** PFASs, Transporter, Renal reabsorption, In vitro, URAT1, OAT4

## Abstract

Poly- and perfluoroalkyl substances (PFASs) are omnipresent in the environment and have been shown to accumulate in humans. Most PFASs are not biotransformed in animals and humans, so that elimination is largely dependent on non-metabolic clearance via bile and urine. Accumulation of certain PFASs in humans may relate to their reabsorption from the pre-urine by transporter proteins in the proximal tubules of the kidney, such as URAT1 and OAT4. The present study assessed the in vitro transport of 7 PFASs (PFHpA, PFOA, PFNA, PFDA, PFBS, PFHxS and PFOS) applying URAT1- or OAT4-transfected human embryonic kidney (HEK) cells. Virtually no transport of PFASs could be measured in URAT1-transfected HEK cells. All PFASs, except PFBS, showed clear uptake in OAT4-transfected HEK cells. In addition, these in vitro results were further supported by in silico docking and molecular dynamic simulation studies assessing transporter–ligand interactions. Information on OAT4-mediated transport may provide insight into the accumulation potential of PFASs in humans, but other kinetic aspects may play a role and should also be taken into account. Quantitative information on all relevant kinetic processes should be integrated in physiologically based kinetic (PBK) models, to predict congener-specific accumulation of PFASs in humans in a more accurate manner.

**Supplementary Information:**

The online version contains supplementary material available at 10.1007/s00204-022-03428-6.

## Introduction

Per- and polyfluoroalkyl substances (PFASs) are persistent organic pollutants that are omnipresent in the environment and that have been shown to accumulate in humans (Pizzurro et al. 2019). They contain one or more fluoro-carbon chain with varying chemical groups attached, giving them unique chemical and physical characteristics, such as oil and water repellency, high temperature and chemical resistance, and emulsifying/surfactant properties. PFASs are widely used in various industrial and consumer applications, such as firefighting foams, electronics, textiles, food contact materials, and cosmetics. According to OECD (2018), over 4700 PFASs have been identified. The production and use of the best-known and most-studied PFASs, perfluorooctanoic acid (PFOA) and perfluorooctane sulfonate (PFOS), have been restricted globally due to risk concerns for human health and the environment (EU 2019, 2020; UNEP 2009).

A critical concern related to PFASs is their persistence both in humans and the environment. In general, PFASs are not biotransformed in animals and humans, so that elimination is dependent on non-metabolic clearance. Pizzurro et al. (2019) collected kinetic data of several PFASs (PFBA, PFOA, PFBS, PFHxS, PFOS) in different species (rats, mice, rabbits, monkeys and humans) and observed notable differences amongst the different PFASs regarding species- and substance-specific tissue partitioning, half-life, and transfer to offspring via the placenta or lactation. Pizzurro et al. (2019) concluded that regarding excretion, half-lives are longer for the eight-carbon versus the four-carbon PFASs and longer for the sulfonates compared to the carboxylates. Humans were found to have much longer half-lives than the animals, especially for PFOA, PFHxS and PFOS (Pizzurro et al. 2019). PFASs have been reported to be eliminated via bile and urine, but excretion for PFASs in humans is in general considered to be limited, causing accumulation after repeated (daily) exposure. Extensive binding to plasma proteins such as albumin (e.g. see overview in Han et al. 2012) and enterohepatic recirculation (Fujii et al. 2015) may play a role in the long residence time of PFASs in the body. Another reason underlying the limited elimination of PFASs are transporters expressed in the kidney, actively reabsorbing PFASs from the pre-urine into the proximal tubule cells of the kidney and subsequent reuptake in the blood stream (Han et al. 2012; Pizzurro et al. 2019).

Given that transporters play a major role in the elimination kinetics of PFASs and since large species differences exist in expression and activity of transporters in the kidney, allometric scaling of animal kinetic data on PFAS elimination (Kim et al. 2019) is not an adequate approach to estimate PFAS elimination in humans (Hethey et al. 2019, 2020). In humans, OAT4 and URAT1 are considered important transporters in the reuptake of PFASs from pre-urine (Yang et al. 2010; Nakagawa et al. 2009). PFOA has been shown to be a substrate for OAT4 (OAT4-expressing CHO cells: *K*_m_: 310 µM; *V*_max_: 37 nmol min^−1^ mg protein^−1^); Yang et al. 2010) and for URAT1 (URAT1-expressing HEK293 cells: *K*_m_: 64 µM; *V*_max_: 0.32 nmol min^−1^ mg protein^−1^; Yang et al. 2010), suggesting a major role for OAT4 in the reabsorption of PFOA. For PFOA and a series of other PFASs, estrone-3-sulphate and uric acid inhibition studies were performed and showed that PFOA and other PFASs decreased estrone-3-sulphate and uric acid uptake, indicating that also other PFASs may interact with these reuptake transporters in the kidney. However, kinetic datasets on OAT4- and/or URAT1-mediated transport of other PFASs than PFOA are currently lacking.

**Fig. 1.**
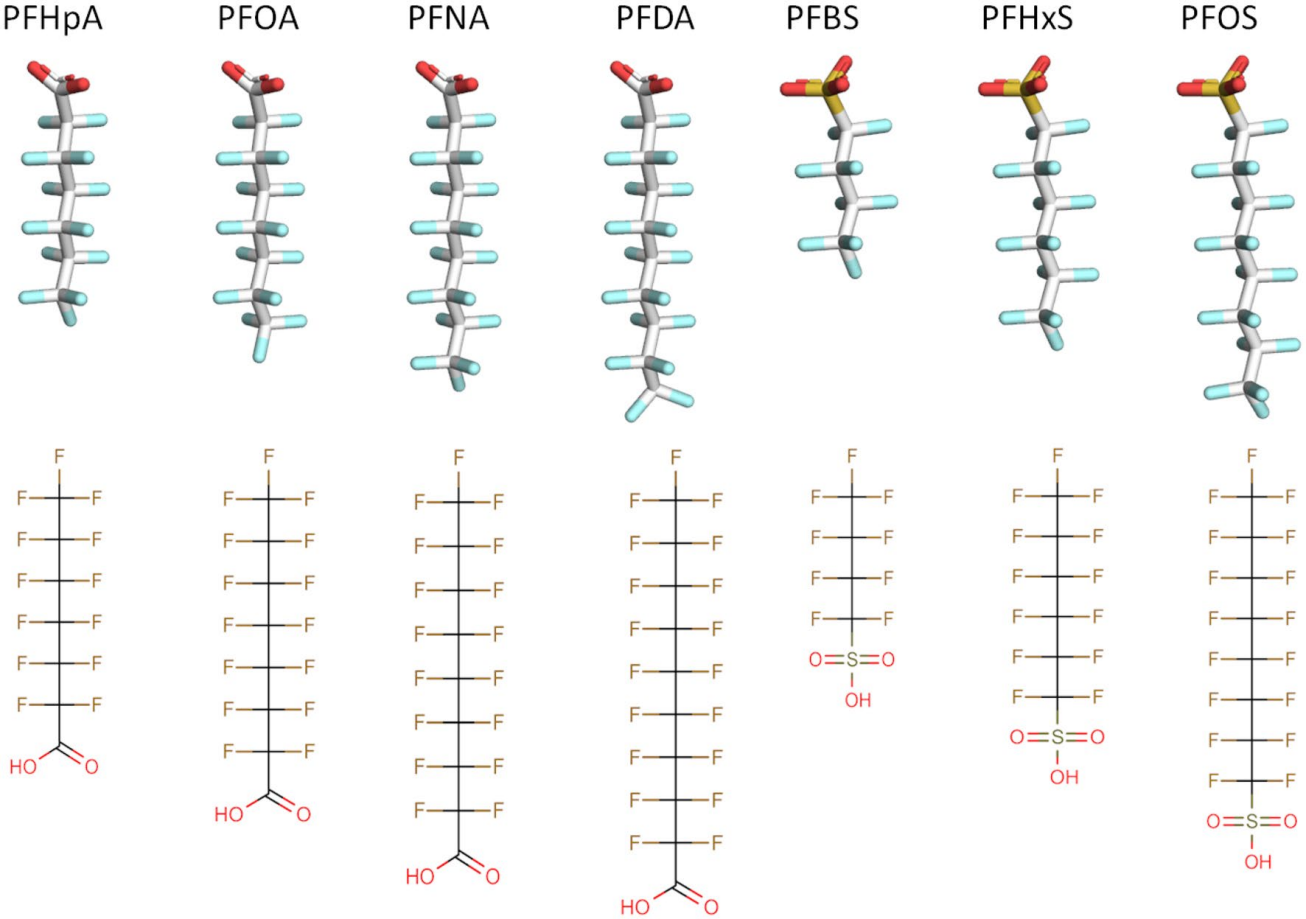
3D and 2D representation of PFASs investigated in the present study

To improve our understanding of kinetic processes of PFASs in humans, in vitro and in silico studies on the interaction with transporters are essential. The present study aims to determine transporter kinetics of a series of PFASs (Fig. 1) for human OAT4 and human URAT1. To that end, cell accumulation studies were performed with transporter-transfected human embryonic kidney (HEK) cells and control-transfected cells. For PFASs that showed a clear (> twofold) increase in cellular accumulation in transporter-transfected cells compared to control-transfected cells, full kinetic studies were performed, and apparent *K*_m_, *V*_max_ and transporter efficiency (*V*_max_/*K*_m_) values were derived from the obtained data. In addition, in silico docking and molecular dynamic simulation studies were performed to characterise transporter–ligand interactions to provide a mechanistic rationale for the measured in vitro evidence.

## Materials and methods

### Test chemicals

Perfluoroheptanoic acid (PFHpA, 99% purity), perfluorooctanoic acid (PFOA, 95% purity), perfluorononanoic acid (PFNA, 99% purity), perfluorodecanoic acid (PFDA, 98% purity) and perfluorobutane sulfonate (PFBS; 97% purity) were obtained from Sigma-Aldrich (Zwijndrecht, The Netherlands). Perfluorohexane sulfonate (PFHxS, 95% purity) and perfluorooctane sulfonate (PFOS, 97% purity) were obtained from Synquest laboratories (Alachua FL, USA). All stocks were prepared in 100% dimethyl sulfoxide (DMSO HybriMax, Sigma-Aldrich). Final solvent concentrations in studies amounted to 0.1%.

### In vitro kinetic studies

#### In vitro transport studies

The recombinant baculoviruses that contain the cDNA of human transporters OAT4 and URAT1 downstream of a cytomegalovirus (CMV) promotor were generated as previously described (Smeets et al. 2020). As a negative control, baculovirus containing the gene for enhanced yellow fluorescence protein (eYFP) was produced similarly. Moreover, transduction of HEK293 cells and uptake assays were essentially performed as previously described (Smeets et al. 2020). The culture medium was removed and cells were washed with buffer (HBSS plus 10 mM HEPES, pH 7.4). Uptake was initiated by replacing this solution with 150 µL of fresh transport buffer (HBSS (Life Technologies Invitrogen (Carlsbad, CA, USA))-HEPES (Sigma-Aldrich), pH 7.4) supplemented with the PFASs for 10 min. To terminate the reaction, transport buffer was replaced with 400 µL ice-cold HBSS-HEPES buffer containing 0.5% BSA (m/v; Roche Diagnostics GmbH (Mannheim, Germany)), after which the cells were washed twice with ice-cold HBSS-HEPES. Cellular PFAS concentrations were determined based on the method described by Rosenmai et al. (2018). Briefly, cells were solubilised in 250 µL lysis buffer and cell lysates were stored in Eppendorf tubes at − 80 °C and defrozen when needed for LC–MS analysis and for quantification of the protein concentration.

#### Sample preparation and LC–MS analysis

To 50 μL cell lysate, 850 μL methanol (Actu-All Chemicals, Oss, The Netherlands) containing internal standards (^13^C_4_-PFHpA, ^13^C_4_-PFOA, ^13^C_5_-PFNA, ^13^C_2_-PFDA, ^13^C_3_-PFBS, ^18^O_2_-PFHxS and ^13^C_4_-PFOS (Wellington Laboratories, Canada) was added. These dilutions were vortexed well before centrifugation at maximum speed for 10 min at 4 °C. PFAS concentrations in the supernatant were determined using LC–MS/MS analysis. If required, samples were further diluted with methanol and containing internal standards. LC–MS/MS analysis was based on a Shimadzu UHPLC system containing: 2 pumps (LC 20AD xr); column oven (Shimadzu CTO-20AC); pump switch (Shimadzu FCV-11AL); degasser (Shimadzu DGU-20A3); and sample tray holder (Shimadzu SIL-20 AC XR model) (Shimadzu Corporation, Kyoto, Japan). An Acquity BEH-C18 analytical column (50 × 2.1 mm i.d., 1.7 μm, Waters, Milford, MA, USA) was used to separate the PFASs at a column temperature of 35 °C. In addition, a symmetry C18 analytical column (50 × 2.1 mm i.d., 5 µm, Waters, Milford, MA, USA) was used as a guard column, placed between the pump and the injector valve to isolate and delay interferences out of the LC system. The mobile phase consisted of 2 mM ammonium acetate (Merck Millipore, Darmstadt, Germany) in Milli-Q water (prepared using a Milli-Q system with a resistivity of at least 18.2 MΩ cm^−1^ (Merck Millipore)) (mobile phase A) and Acetonitrile ULC/MS grade (Actu-All Chemicals, Oss, The Netherlands) (mobile phase B). The injection volume used was 20 μL. The chromatographic gradient was operated at a flow rate of 0.3 mL min^−1^ starting from 25% mobile phase B in the first 0.1 min, a linear increase to 100% B in 6 min with a final hold of 2.5 min. The gradient was returned to 25% B within 0.1 min for 3.9 min to equilibrate before the next injection, resulting in a total run of 12.5 min.

Detection was carried out by MS/MS using a Sciex QTRAP 5500 system (Sciex, Framingham, MA, USA) in negative electrospray ionisation (ESI-) mode, with the following conditions: ion spray voltage (IS) of − 4500 V; curtain gas (CUR) of 30 L h^− 1^; source temperature (TEM) of 350 °C; gas 1 (GS1) of 55 L h^−1^; gas 2 (GS2) of 60 L h^−1^; and collision gas (CAD) high. The PFASs were fragmented using collision-induced dissociation (CID) using argon as target gas. The analyses were performed in multiple reaction monitoring (MRM) mode, using two mass transitions per component selected based on the abundance of the signal and the selectivity of the transition. In Supplementary Table 1, information on the MRM transitions, de-clustering potential (DP), entrance potential (EP), collision energy (CE) and cell exit potential (CXP) are presented. Data were acquired using Analyst software and processed using MultiQuantTM software (Sciex, Framingham, MA, USA).

#### Protein concentration determination

Cellular PFAS content was normalised to cellular protein. To that end, from each cell lysate, 25 µL was used to determine the protein concentration (technical duplicate) using the Pierce BCA Protein Assay Kit (Thermo Scientific, Waltham, MA). First, samples were diluted two times with lysis buffer directly in a 96-wells plate (PS, F-bottom, clear; Greiner Bio-One, Alphen aan de Rijn, The Netherlands), and in each plate, a BSA standard curve (0.05–2 mg mL^−1^ in lysis buffer) was included allowing protein quantification of the samples. The manufacturer’s protocol was followed for the subsequent steps and absorbance was measured using a microplate reader (Synergy™ HT BioTek, Winooski, VT) at 562 nm.

### In silico molecular modelling studies

#### Data source

The set of low-molecular weight molecules analysed in this work included prostaglandin E2, uric acid, PFHpA, PFOA, PFNA, PFDA, PFBS, PFHxS and PFOS. The structures were retrieved in the structure-data file (.sdf) format from the PubChem database (https://pubchem.ncbi.nlm.nih.gov) (Kim et al. 2021) with the following respective PubChem CID: 5280360, 1175, 67818, 9554, 67821, 9555, 67815, 67734 and 74483. The primary sequence of human OAT4 (Solute carrier family 22 member 11) and URAT1 (Solute carrier family 22 member 12) was retrieved in the FASTA format from the UniProt database (https://www.uniprot.org) (UniProt Consortium 2021) records with code Q9NSA0 and Q96S37, respectively. The availability of 3D structures for human OAT4 and URAT1 was searched for in the Protein DataBank (PDB; https://www.rcsb.org) (Berman et al. 2000); however, no entries were found (last database access: 7th of July 2021).

### Homology modelling

Homology modelling was applied to derive the 3D structures of human OAT4 and URAT1, as no structures were publicly available at the time of analysis (last access to PDB: 7th of July 2021). To do so, the trRosetta algorithm (https://yanglab.nankai.edu.cn/trRosetta) (Yang et al. 2020) was used with the human sequences of OAT4 and URAT1 in the FASTA format as input, and allowing the search of PDB templates for a better prediction. Overall, trRosetta is an algorithm for a fast and accurate protein structure prediction. It builds the protein structure based on direct energy minimisations with a constrained optimisation by Rosetta (Simons et al. 1999). The restraints include inter-residue distance and orientation distributions, predicted by a deep residual neural network. Homologous templates found in PDB were included in the network prediction to improve the accuracy of the predicted 3D structures.

#### Model and ligands preparation

The consistency of all atom and bond type assignment and geometries of ligands and transporter models was visually checked with UCSF Chimera software (version 1.15) (Pettersen et al. 2004). All the carboxylic groups of ligands were deprotonated as expected under physiological conditions and the 3D structures were saved as Tripos MOL2 molecule file (.mol2) for subsequent analysis. Transporter models were further relaxed and energetically minimised to avoid steric clashes and to correct any eventual improper structural geometries by means of GROMACS (Abraham et al. 2015) with CHARMM27 all-atom force field parameters support (Best et al. 2012). In particular, the steepest descent minimisation algorithm was used with an energy step size of 0.01, a maximum allowed number of steps set at 5000 and an energy threshold to stop minimisation set at 10.0 kJ mol^−1^.

#### Docking analysis

The docking analysis was performed using the AutoDock Vina software (version 1.1.2) (Trott and Olson 2010), graphically interfaced in the UCSF Chimera software (version 1.15) (Pettersen et al. 2004). The binding site was defined within a box (15 × 15 × 15 Å; *L* × *W* × *H*) centred at the channel entrance. Default parameters were applied, except for the number of binding modes that were set at 1, the research exhaustiveness set at 4 and the maximum energy difference set at 3 kcal mol^−1^. Each docking simulation was run in triplicate to account for fluctuations due to the genetic algorithm implemented in VINA, and scores are expressed as means ± standard deviation in agreement with previous studies (Aichinger et al. 2020). Once the stability of score assignment and binding architecture were ascertained, the best scored pose for each ligand was carried forth to the analysis, in agreement with previous studies (Aichinger et al. 2020).

#### Molecular dynamic simulation

Molecular dynamic simulations were performed to investigate the overall geometrical stability of OAT4- and URAT1-ligand complexes over time. Molecular dynamic simulations were performed using GROMACS (version 5.1.4) (Abraham et al. 2015) with CHARMM27 all-atom force field parameters support (Best et al. 2012) and using the best score docking position as starting position for all ligands. All the ligands have been processed and parameterised with CHARMM27 all-atom force field using the SwissParam tool (http://www.swissparam.ch) (Zoete et al. 2011). Input protein–ligand complexes were solvated with SPCE waters in a cubic periodic boundary condition, and counter ions (Na^+^ and Cl^−^) were added to neutralise the system. Prior to Molecular dynamic simulation, the systems were energetically minimised to avoid steric clashes and to correct improper geometries using the steepest descent algorithm with a maximum of 5000 steps. Afterwards, all the systems underwent isothermal (300 K, coupling time 2 ps) and isobaric (1 bar, coupling time 2 ps) 100 ps simulations before running an early-term 8 ns simulation, to monitor the earliest induced-fit movements (300 K with a coupling time of 0.1 ps and 1 bar with a coupling time of 2.0 ps).

## Results

### In vitro kinetic studies

**Fig. 2:**
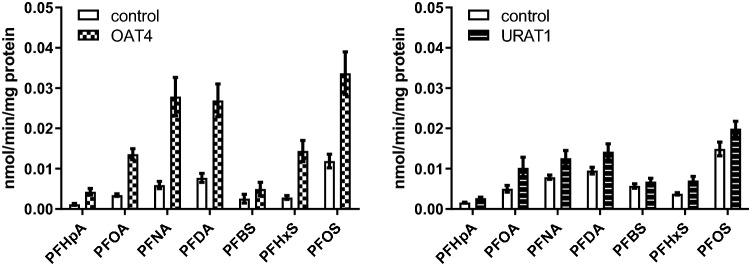
Cellular PFAS levels in transporter-transfected cells (OAT4 or URAT1) or control-transfected cells (no transporter) upon exposure of cells to a mixture of PFASs (1 µM, for 10 min). Data are shown as the average (bars) and standard error of the mean (error bars) of six replicates from two studies (three replicates per study) per condition

In a first study, PFAS congeners substantially transported by OAT4 or URAT1 were identified to select those considered most relevant to be further analysed in detail. To that end, transporter-transfected cells and control-transfected cells (no transporter) were exposed to a mixture of the PFASs with each congener applied at 1 µM for 10 min. The results of these two studies are presented in Fig. 2 and substantial transport was concluded when cellular PFAS concentration was at least twofold higher in transporter-transfected cells compared to that in control-transfected cells. Overall, substantial transport with OAT4 was concluded for PFHpA, PFOA, PFNA, PFDA, PFHxS and PFOS but not for any of the congeners with URAT1 (Fig. 2). Also when applying a tenfold higher concentration (10 µM), no appreciable transport of PFASs by URAT1 was detected (Supplementary Fig. 1).

**Fig. 3:**
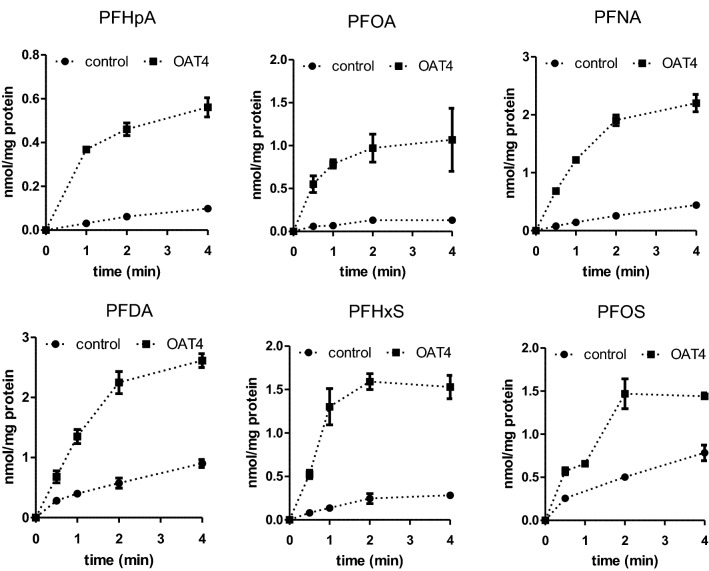
Cellular PFAS levels in transporter-transfected cells (OAT4) or control-transfected cells (no transporter) upon exposure of cells to PFHpA, PFOA, PFNA, PFDA, PFHxS or PFOS, each at 10 µM, for different exposure durations. Data are shown as the average (square) and standard error of the mean (error bars) of three replicates per condition

For those PFASs that showed substantial transport, the time-dependent cellular accumulation in transporter-transfected cells and control-transfected cells was then investigated upon exposure to each PFAS (single exposure) at a concentration of 10 µM and results are presented in Fig. 3. These data were used to determine an adequate time-point to perform the full kinetic studies to characterise the transport of the PFASs by OAT4. The authors considered 1 min exposure as the most accurate time-point for exposure duration, being in general in the linear part of the cellular uptake feasible to perform the incubations and providing data that can be reliably quantified.

**Fig. 4:**
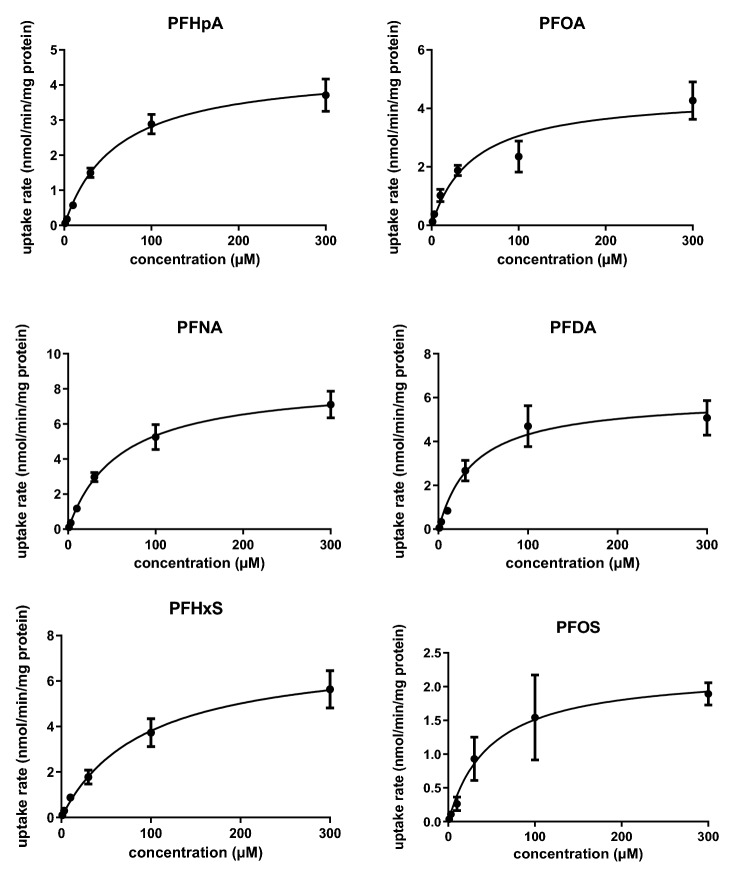
OAT4-mediated cellular uptake rates of PFHpA, PFOA, PFNA, PFDA, PFHxS and PFOS. OAT4-transfected cells and control-transfected cells were exposed for 1 min to a range of PFAS concentrations and cellular amounts were determined. Amounts obtained in control-transfected cells were subtracted from amounts obtained in OAT4-transfected cells to obtain OAT4-mediated cellular uptake rates. Data are presented as average (circles) and standard error of the mean (error bars) of 6 data points per condition obtained from 3 studies. The Michaelis–Menten equation was fit to the data using Graphpad Prism version 9.2.0 and related kinetic constants (apparent *K*_m_ and *V*_max_ and transporter efficiencies) are presented in Table 1

In a next step, the Michaelis–Menten kinetics for OAT4-mediated cellular uptake of PFHpA, PFOA, PFNA, PFDA, PFHxS and PFOS was determined. OAT4- and control-transfected cells were exposed for 1 min to a range of PFAS concentrations (1, 3, 10, 30, 100 and 300 µM) and cellular amounts were determined. Quantities as congener amounts obtained from control-transfected cells were subtracted from those obtained in OAT4-transfected cells for each condition and resulting data are presented in Fig. 4. Data were subsequently used to estimate the apparent *K*_m_ and *V*_max_ of the reactions and the related transporter efficiency values (*V*_max_/*K*_m_) as illustrated in Table 1. From these studies, apparent *K*_m_ values ranged between 39 µM (PFDA) and 92 µM (PFHxS) and apparent *V*_max_ values ranged between 2.2 nmol min^−1^ mg protein^−1^ (PFOS) and 8.5 nmol min^−1^ mg protein^−1^ (PFNA). Transporter efficiencies ranged between 46 µL min^−1^ mg protein^−1^ (PFOS) and 154 µL min^−1^ mg protein^−1^ (PFDA).

**Table 1:** Apparent *K*_m_ and *V*_max_ values and related transporter efficiencies (*V*_max_/*K*_m_) of OAT4-mediated cellular uptake of PFHpA, PFOA, PFNA, PFDA, PFHxS and PFOS obtained in the present study

	Apparent* K*_m_ (µM)	Apparent *V*_max_ (nmol min^−1^ mg protein^−1^)	Transporter efficiency (µL min^−1^ mg protein^−1^)	Reported albumin/water partition coefficients (L kg^−1^)^a^	Reported half-life in humans (years)
Perfluoroalkyl carboxylic acids (PFCAs)					
PFHpA (C7)	60 (15)	4.5 (0.4)	75	4.0	0.17^b^
PFOA (C8)	47 (15)	4.5 (0.5)	96	4.2	1.8–3.8^c^
PFNA (C9)	58 (15)	8.5 (0.7)	147	4.3	
PFDA (C10)	39 (15)	6.0 (0.7)	154	4.7	
Perfluoroalkyl sulfonic acids (PFSAs)					
PFBS (C4)	NA	NA	NA	3.2	0.07–0.12^d^
PFHxS (C6)	92 (33)	7.3 (1.0)	79	4.8	2.9–8.5^c^
PFOS (C8)	48 (24)	2.2 (0.4)	46	4.7	1.8–5.4^c^

Data obtained from three studies (Fig. 4) were analysed using Graphpad Prism version 9.2.0 and are presented as best-fit values and standard errors between brackets. For comparison, reported albumin/water partition coefficients and half-lives in humans are also included

^a^Taken from Allendorf et al. (2019)

^b^From Xu et al. (2020)

^c^From Olsen et al. (2007), Li et al. (2018), and Xu et al. (2020)

^d^From Olsen et al. (2009)

### In silico molecular modelling studies

In a previous in silico study on the interaction of chemicals with OAT1, it was shown that 3D molecular modelling can provide a mechanistic basis to discriminate transporter substrates from chemicals that are not (substantially) transported (decoys) (Louisse et al. 2022). Specifically, straightforward geometrical properties, including the detection of interaction with the inner part of the transporter’s funnel and monitoring the very early inward trajectories of ligands, provide geometrical parameters to discriminate substrates from non-substrate molecules. In the present study, this in silico approach was applied to provide more insight into the transport of PFASs by OAT4 and URAT1. The interactions of PFASs with OAT4 and URAT1 transporters and the ligand movements were analysed using docking studies and molecular dynamic simulations, respectively. Specifically, docking simulations ware done in triplicates to account for the variability of docking outcomes due to the genetic algorithm implemented in the docking software (see Materials and methods section for further details). Both the score assignment and the binding architecture were found consistent amongst the replicates for each ligand (Table 2; Supplementary Table 2 and Supplementary Fig. 2). However, docking scores could not discriminate transporter substrates from molecules not efficiently transported, as identified in vitro (Table 2). This result was in agreement with previous evidence from a study dealing with a closely related paralogue transporter (i.e. OAT1) (Louisse et al. 2022). In that study, Louisse et al. demonstrated that docking scores could not discriminate the set of true ligands (i.e. transporter substrates) from true decoys (i.e. non-substrate molecules), whilst the placement of binding architecture in respect to the channel funnel and further molecular dynamics effectively discriminated substrates from reported non-ligands (Louisse et al. 2022). Consistently with this observation, the analysis of binding architectures and their dynamics provided a consistent line of evidence to corroborate the experimental in vitro data obtained here.

**Table 2:** Docking scores of PFASs and probe substrates of URAT1 and OAT4

Compound	OAT4		URAT1	
	Score (kcal mol^−1^; mean ± standard deviation)^a^	Substrate	Score (kcal mol^−1^; mean ± standard deviation)^a^	Substrate
PFHpA	− 6.1 ± 0.0	Yes	− 5.5 ± 0.0	No
PFOA	− 6.2 ± 0.0	Yes	− 6.0 ± 0.0	No
PFNA	− 6.7 ± 0.2	Yes	− 6.6 ± 0.0	No
PFDA	− 7.1 ± 0.0	Yes	− 6.7 ± 0.0	No
PFHxS	− 6.4 ± 0.0	Yes	− 5.9 ± 0.1	No
PFOS	− 7.2 ± 0.2	Yes	− 6.9 ± 0.1	No
PFBS	− 5.5 ± 0.0	No	− 4.9 ± 0.0	No
Uric acid	–	–	− 6.6 ± 0.0	Yes
Prostaglandin E2	− 6.0 ± 0.0	Yes	–	–

^a^The scores are reported as means of three independent runs ± standard deviation

**Fig. 5:**
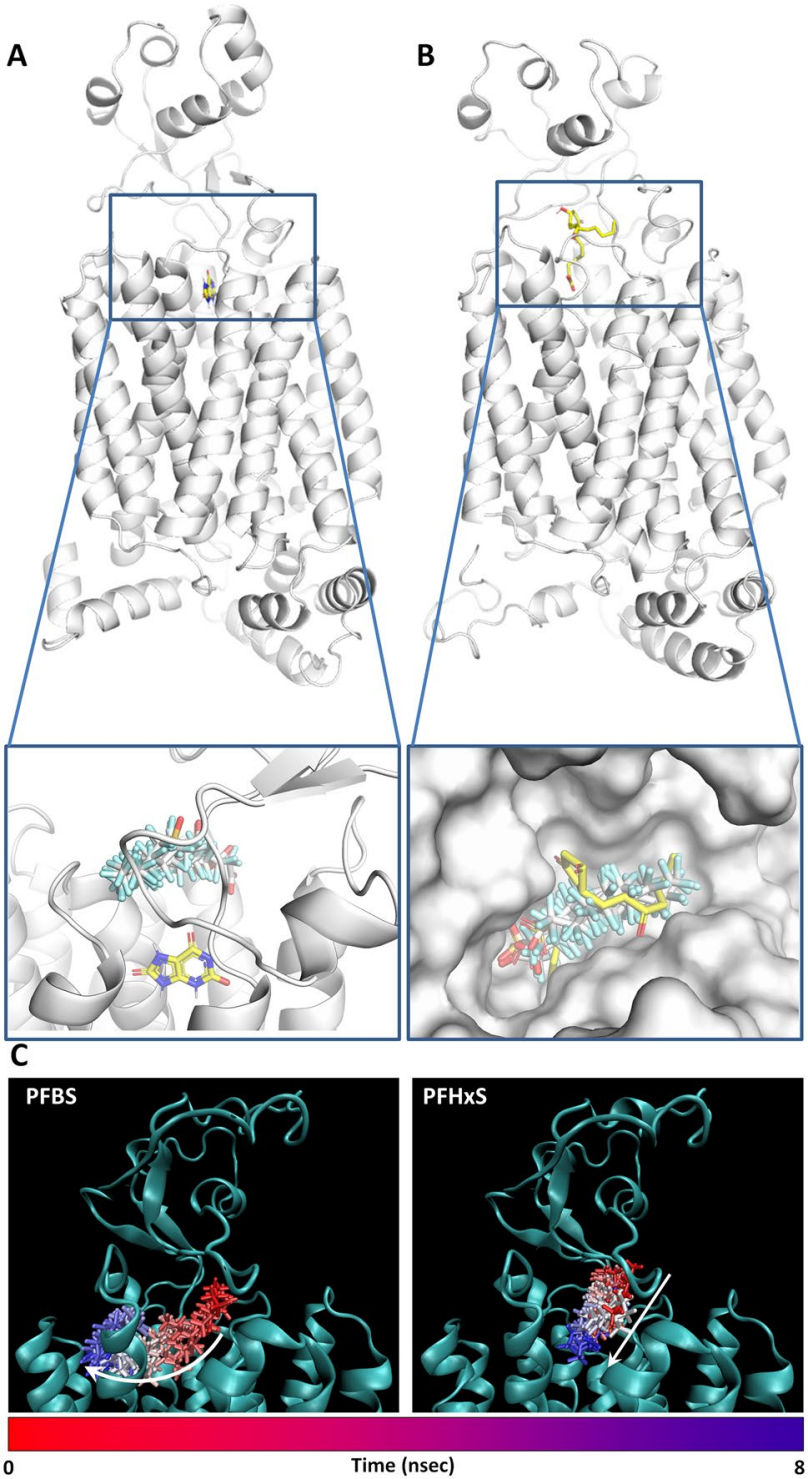
Computational results. **A** Binding architecture of PFASs and uric acid within URAT1. The whole protein is represented in cartoon and uric acid in yellow sticks. In the close-up detailing the binding site, PFASs are represented in white sticks, and uric acid in yellow sticks. **B** Binding architecture of PFASs and prostaglandin E2 within OAT4. The whole protein is represented in cartoon, whilst prostaglandin E2 is represented in yellow sticks. In the close-up detailing the binding site, PFASs are represented in white sticks, prostaglandin E2 in yellow sticks and protein is represented in white surface for a better view of pocket occupancy. **C** Molecular dynamics results of PFBS and PFHxS for OAT4. The from-red-to-blue colour switch indicates the stepwise changes of ligands coordinates along the simulation. The white arrows indicate the trajectory of ligands

As shown in Fig. 5A and B, none of the PFASs investigated here were found to interact with the inner part of URAT1 like uric acid, which was selected as URAT1’s probe substrate (Iwanaga et al. 2005; Morrissey et al. 2012). Therefore, none of the PFASs included in this study provided an architecture of binding likely prone for being transported efficiently by URAT1. With regard to OAT4, all the PFASs adopted an orientation similar to that of prostaglandin E2, selected as the reference compound for high affinity OAT4 substrates (Kimura et al. 2002; Morrissey et al. 2012). On this basis, an efficient transport by OAT4 could be expected for all of them. However, initial screening uptake experiments showed that PFBS transport is rather limited compared to other PFASs tested in this study (Fig. 2). Although the docking score of PFBS suggests a lower stability of the protein–ligand complex (the more negative the score, the more stable the expected stability of the protein–ligand complex), we showed previously that the docking score itself was not able to discriminate between substrates and decoys of OAT1 (Louisse et al. 2022). The existence of other mechanisms, downstream from the initial PFBS binding, was hypothesised and analysed through molecular dynamic simulations. Specifically, the trajectory of PFBS was compared to that of the closely related congener PFHxS, which showed an appreciable transport in in vitro uptake studies (Figs. 2, 3, and 4) and for which the docking score was closest to that of PFBS. As shown in Fig. 5C, PFHxS drew a clear inward trajectory. Conversely, the trajectory of PFBS described a lateral sliding along the surface of the transporter mouth, rather than an inward movement, possibly resulting in a protein–ligand detachment. These movements are better explained in the movies (see Supporting Materials) drawing the top view of the two complexes where the capability of PFHxS to stay properly arranged at the protein funnel and the lateral sliding of PFBS are dynamically represented. Therefore, the lack of an appreciable transport for PFBS found experimentally could be explained by a mechanistic event later than the transporter recognition. The very low number of carbons in the PFBS chain compared to the other PFASs analysed here may play a role, where the compound is not prone for being directed towards the inner part of the protein to allow transport, but rather moved away from the transporter.

## Discussion

The present study aimed to characterise the transport of a series of PFASs (PFHpA, PFOA, PFNA, PFDA, PFBS, PFHxS and PFOS) by human OAT4 and URAT1. These data are useful to obtain more insights into the possible carrier-mediated reabsorption of these chemicals from the pre-urine into the circulation by the kidney, expected to play a role in the accumulation and long half-lives of these chemicals in humans. In vitro transport studies with PFAS mixtures with human OAT4- and URAT1-transfected HEK293 cells showed a more than twofold increase in cellular uptake in OAT4-transfected cells for PFHpA, PFOA, PFNA, PFDA, PFHxS and PFOS compared to control cells. Full kinetic studies for these PFASs revealed a maximum 3.3-fold difference in transporter efficiency (*V*_max_/*K*_m_) between the compounds, with the most efficient OAT4-mediated transport for PFDA and least efficient OAT4-mediated transport for PFOS. The transport studies pointed to limited transport of all tested PFASs by URAT1 and for PFBS by OAT4. Results from the in vitro kinetic studies were supported by the results from the in silico modelling studies.

Previous reports have shown PFOA to be a substrate for OAT4 and a selection of other PFASs as inhibitors of OAT4-mediated estrone-3-sulphate uptake, indicating that these PFASs may also interact with OAT4 (Yang et al. 2010). The present study shows, that besides PFOA, PFHpA, PFNA, PFDA, PFHxS and PFOS are also OAT4 substrates and demonstrated limited congener-specific differences in transporter kinetics, exemplified by differences in transporter efficiencies between 46 and 154 µL min^−1^ mg protein^−1^. From initial screening studies, no substantial PFAS transport by URAT1 in URAT1-transfected HEK cells could be demonstrated (i.e. < two fold increase in cellular accumulation in URAT1-transfected cells compared to control-transfected cells). URAT1-mediated PFOA transport has been reported before by Yang et al. (2010), but the transporter efficiency for URAT1-mediated transport was 24-fold lower than that for OAT4-mediated transport (Yang et al. 2010), suggesting that OAT4 may be more important than URAT1 in mediating the renal reabsorption of PFOA. Data from the present study support this hypothesis that OAT4 is likely to be more important in PFOA reabsorption than URAT1 and suggests this to be also the case for PFHpA, PFNA, PFDA, PFHxS and PFOS.

Novel approach methods (NAMs), including in vitro and in silico kinetic studies, as performed in the present study, can provide useful information for the hazard assessment of chemicals, particularly to assess whether a chemical is expected to accumulate in humans, and as such of possible concern. From this perspective, it is of interest to compare information on reported half-lives of PFASs in humans with data obtained in this study on OAT4 transporter kinetics. Of the tested PFASs in the present study, serum half-lives in humans have been reported for PFHpA (Xu et al. 2020), PFOA (Olsen et al. 2007; Li et al. 2018; Xu et al. 2020), PFBS (Olsen et al. 2009), PFHxS (Olsen et al. 2007; Li et al. 2018; Xu et al. 2020) and PFOS (Olsen et al. 2007; Li et al. 2018; Xu et al. 2020). Reported (average) serum half-lives (Table 1) are shortest for PFBS (26 to 44 days) and PFHpA (62 days). The reported (average) serum half-lives of the other PFASs are longer, ranging from 1.8 to 3.8 years for PFOA, 1.8 to 5.4 years for PFOS, and 2.9 to 8.5 years for PFHxS. A comparison of the reported in vivo half-lives in humans for PFHpA, PFOA, PFBS, PFHxS and PFOS (0.17, 1.8 to 5.4, 0.07 to 0.12, 2.9 to 8.5 and 1.8 to 5.4 years, respectively) with the transporter efficiencies of OAT4-mediated transport obtained in this study (75, 96, not determined, 79 and 46 µL min^−1^ mg protein^−1^, respectively) indicated that no direct correlation between these two parameters exists. The short half-life of PFBS is in line with the finding that PFBS is hardly transported by OAT4. Notwithstanding, other kinetic processes determine the elimination of PFASs from the body, besides OAT4-mediated resorption in the kidney, and these may also show congener-specific differences, as further discussed in the next section. However, it is of interest to note that when splitting the group of PFASs in perfluoroalkyl carboxylic acids (PFHpA and PFOA) and perfluoroalkyl sulfonic acids (PFBS, PFHxS and PFOS), the transporter efficiencies correctly predicted shorter half-lives for PFHpA compared to PFOA and for PFHxS compared to PFOS (and for both shorter compared to PFBS). Although these findings are based on a limited number of congeners and do not necessarily translate to other perfluoroalkyl carboxylic acids or perfluoroalkyl sulfonic acids, it can be hypothesised that based on the transporter efficiencies of OAT4 transport obtained in the present study, the half-lives of PFNA and PFDA would be even longer in humans than that of PFOA.

Renal elimination of PFASs does not solely depend on reabsorption from pre-urine to the circulation by transporters like OAT4. Differences in elimination may also relate to differences in plasma protein binding and related differences in excretion via glomerular filtration, as well as possible differences in active secretion via transporter proteins. Of the PFASs tested in the present study, differences in albumin binding (Table 1) have been reported, particularly for PFBS showing a relatively low binding to albumin compared to the other PFASs (Allendorf et al. 2019; Gao et al. 2019; Lu et al. 2021). The relatively short half-life of PFBS in humans may relate to such limited plasma protein binding, related to a higher passive excretion via glomerular filtration, besides the expected limited transport of PFBS by OAT4 as determined in the present study. Besides reabsorption by OAT4 and possible other renal transporters, active secretion of PFASs by the kidney may also occur. Uptake transporters such as OAT1 and OAT3 in the basolateral membrane of the proximal tubular cells have been reported to transport PFASs in vitro, resulting in uptake from the circulation, and possible subsequent transport to the pre-urine by other transporter proteins. Weaver et al. (2010) studied rat Oat1-mediated PFHpA and PFOA transport and rat Oat3-mediated PFOA and PFNA transport, whereas Nakagawa et al. (2008) compared rat Oat1- and Oat3-mediated and human OAT1- and OAT3-mediated transport of PFOA. PFASs that are substrates for these transporters may be expected to show more renal elimination than PFASs that are not substrates for such transporters. Studying PFAS congener-related (differences in) transporter kinetics of OAT1 and OAT3 (and possible other transporters) may, therefore, provide additional insights into the (renal) elimination of PFASs and is, therefore, of interest for future research.

The in silico analysis provided ground to understand the mechanistic basis of the in vitro experimental outcomes, particularly the limited capacity of these PFASs to reach the inner part of URAT1’s funnel as a likely structural rationale for the lack of appreciable uptake by URAT1-expressing cells. Conversely, PFASs that were transported by OAT4 were able to reach the inner part of the transporter’s funnel pointing out the relevance of this feature to discriminate substrates from non-substrate molecules, in line with a previous study on OAT1 (Louisse et al. 2022). The analysis of perfluoroalkyl sulfonic acids revealed that monitoring the protein–ligand complex dynamic is critical to allow discrimination between PFHxS and PFBS (OAT4 substrate and non-substrate, respectively), showing that assessment of the early dynamics of interaction is relevant. In particular, PFBS showed the capability to interact with OAT4 like its substrates, but it was unable to move towards the inner part of the transporter as was observed for PFHxS. Although the analysis focussed on the very early molecular events of OAT4-mediated transport, the method provided important general insights. The use of these approaches to predict PFAS transport by other OATs, in a qualitative manner, will lead to a better understanding of congener and species differences in toxicokinetics of PFASs.

Insight into the (transporter) kinetics of chemicals is crucial for the interpretation of (human) toxicity data. When using human data for hazard assessment, one should assess carefully identified correlations since they do not necessarily indicate a causal relationship. From this perspective, it is of interest to include the implications of transporter kinetics. Steenland et al. (2010) found an association between PFOA and PFOS serum levels with uric acid serum levels, which is considered a risk factor for hypertension and cardiovascular outcomes. Interestingly, uric acid is reabsorbed in the kidney by, amongst others, OAT4 (Lipkowitz 2012; Ekaratanawong et al. 2004). This may indicate that individuals with high activity/expression of OAT4, may show a relatively high reabsorption of both uric acid and PFOA and PFOS (and other OAT4 substrates), indicating that increased uric acid levels are not necessarily the result of an increase in PFOA/PFOS exposure. In addition, Steenland et al. (2010) also highlighted that expression/activity of other transporter proteins may play a role in such associations, such as OAT1 and OAT3. These examples indicate that for a better interpretation of human data linking internal PFAS exposure to adverse health outcomes, knowledge on the kinetics is essential to further assess the causality of observed correlations.

In the most recent EFSA PFAS assessment, internal effect concentrations of PFOA, PFNA, PFHxS and PFOS were related to external dose levels using a modified physiologically based kinetic (PBK) model developed by Loccisano et al. (2011) for PFOA and PFOS whilst assuming similar kinetics between PFNA and PFOA and between PFHxS and PFOS (EFSA CONTAM Panel 2020). With regard to refining the analysis of relationships between internal and external dosimetry at defined exposure scenarios for different PFASs, congener-specific PBK models would be required. In this context, information generated using in vitro methods on transporter kinetics (e.g. related to renal excretion and reabsorption and enterohepatic recirculation), as well as plasma protein binding, are essential to develop such models, particularly when in vivo human kinetic data are scarce. Data obtained in the present study can be applied to parameterise such congener-specific PBK models whilst requiring in vitro to in vivo extrapolation to adequately reflect in vivo physiology.

Altogether, the present study shows that the PFASs PFHpA, PFOA, PFNA, PFDA, PFHxS and PFOS are transported by human OAT4 in vitro, which may play a role in their accumulation and the long half-lives observed in humans. These data, combined with other in vitro*-* and in silico-derived kinetic data, can support the parameterisation of PBK models, which can provide full insight into the disposition of these persistent chemicals and the analysis of causal relationships between reported human exposure and effect data.

## Supplementary Information

Below is the link to the electronic supplementary material.

Supplementary file1 (DOCX 598 KB)

Supplementary file2 (MPEG 6122 KB)

Supplementary file3 (MPEG 6661 KB)

## Data Availability

The datasets generated during and/or analysed during the current study are available from the corresponding author on reasonable request
