# Supplementary material for: Perfluoroalkyl substances (PFASs) are substrates of the renal human organic anion transporter 4 (OAT4)
Source: Arch Toxicol. 2022 Nov 27;97(3):685–96. doi: 10.1007/s00204-022-03428-6 (PMC9968691; doi:10.1007/s00204-022-03428-6)
Supplement: Supplementary file 1 — Supplementary file1 (DOCX 598 KB) [file 204_2022_3428_MOESM1_ESM.docx]

#### Supplementary Table 1. MRM transitions of the PFASs tested in the present study.

| **Name** | **Q1 Mass Da** | **Q3 Mass Da** | **Dwell (msec)** | **DP** | **EP** | **CE** | **CXP** |
| --- | --- | --- | --- | --- | --- | --- | --- |
| ^13^C_4_-PFHpA | 366.9 | 321.8 | 4.0 | -55 | -10 | -12 | -55 |
| ^13^C_4_-PFOA | 416.9 | 371.9 | 4.0 | -40 | -15 | -24 | -19 |
| ^13^C_5_-PFNA | 468.0 | 423.0 | 4.0 | -75 | -10 | -16 | -27 |
| ^13^C_2_-PFDA | 515.0 | 470.0 | 4.0 | -65 | -10 | -16 | -31 |
| ^13^C_3_-PFBS | 301.9 | 79.9 | 4.0 | -90 | -10 | -65 | -21 |
| ^18^O_2_-PFHxS | 403.0 | 84.0 | 4.0 | -30 | -10 | -40 | -8 |
| ^13^C_4_-PFOS | 502.9 | 99.0 | 4.0 | -80 | -5 | -34 | -7 |
| PFHpA | 362.9 | 318.9 | 4.0 | -55 | -10 | -12 | -55 |
| PFHpA | 362.9 | 169.0 | 4.0 | -55 | -10 | -24 | -11 |
| PFOA | 412.9 | 369.1 | 4.0 | -40 | -10 | -14 | -11 |
| PFOA | 412.9 | 169.0 | 4.0 | -40 | -15 | -24 | -19 |
| PFNA | 462.9 | 419.1 | 4.0 | -75 | -10 | -16 | -27 |
| PFNA | 462.9 | 169.0 | 4.0 | -75 | -10 | -26 | -11 |
| PFDA | 512.9 | 469.0 | 4.0 | -65 | -10 | -16 | -31 |
| PFDA | 512.9 | 219.0 | 4.0 | -65 | -10 | -26 | -13 |
| PFBS | 298.9 | 79.9 | 4.0 | -90 | -10 | -75 | -21 |
| PFBS | 298.9 | 98.9 | 4.0 | -90 | -10 | -40 | -21 |
| PFHxS | 398.9 | 80.0 | 4.0 | -110 | -10 | -104 | -17 |
| PFHxS | 398.9 | 98.9 | 4.0 | -110 | -10 | -42 | -15 |
| PFOS | 498.9 | 99.0 | 4.0 | -80 | -5 | -94 | -7 |
| PFOS | 498.9 | 80.0 | 4.0 | -80 | -5 | -100 | -11 |

| Supplementary Table ***2***. RMSD analysis of docking replicates for each ligand in OAT4 and URAT1. | | | |
| --- | --- | --- | --- |
| **Protein** | **Ligand** | **RMSD (Å) pose 1 vs pose 2** | **RMSD (Å) pose 1 vs pose 3** |
| OAT4 | PFHpA | 0.003 | 0.010 |
|  | PFOA | 0.757 | 0.026 |
|  | PFNA | 1.329 | 0.930 |
|  | PFDA | 0.707 | 0.200 |
|  | PFBS | 0.054 | 0.897 |
|  | PFHxS | 0.760 | 0.034 |
|  | PFOS | 1.972 | 1.489 |
|  | Prostaglandine E2 | 2.116 | 1.094 |
| URAT1 | PFHpA | 0.007 | 0.802 |
|  | PFOA | 0.759 | 0.010 |
|  | PFNA | 0.707 | 1.064 |
|  | PFDA | 0.889 | 1.414 |
|  | PFBS | 0.901 | 0.004 |
|  | PFHxS | 1.378 | 1.476 |
|  | PFOS | 1.162 | 1.708 |
|  | Uric acid | 0.001 | 0.001 |

#### Supplementary Figure 1. Cellular PFAS levels in transporter-transfected cells (URAT1) or control-transfected cells (no transporter) upon exposure of cells to a mixture of PFASs (10 µM, for 10 minutes). Data are shown as the average (bars) and SEM (error bars) of three replicates per condition.


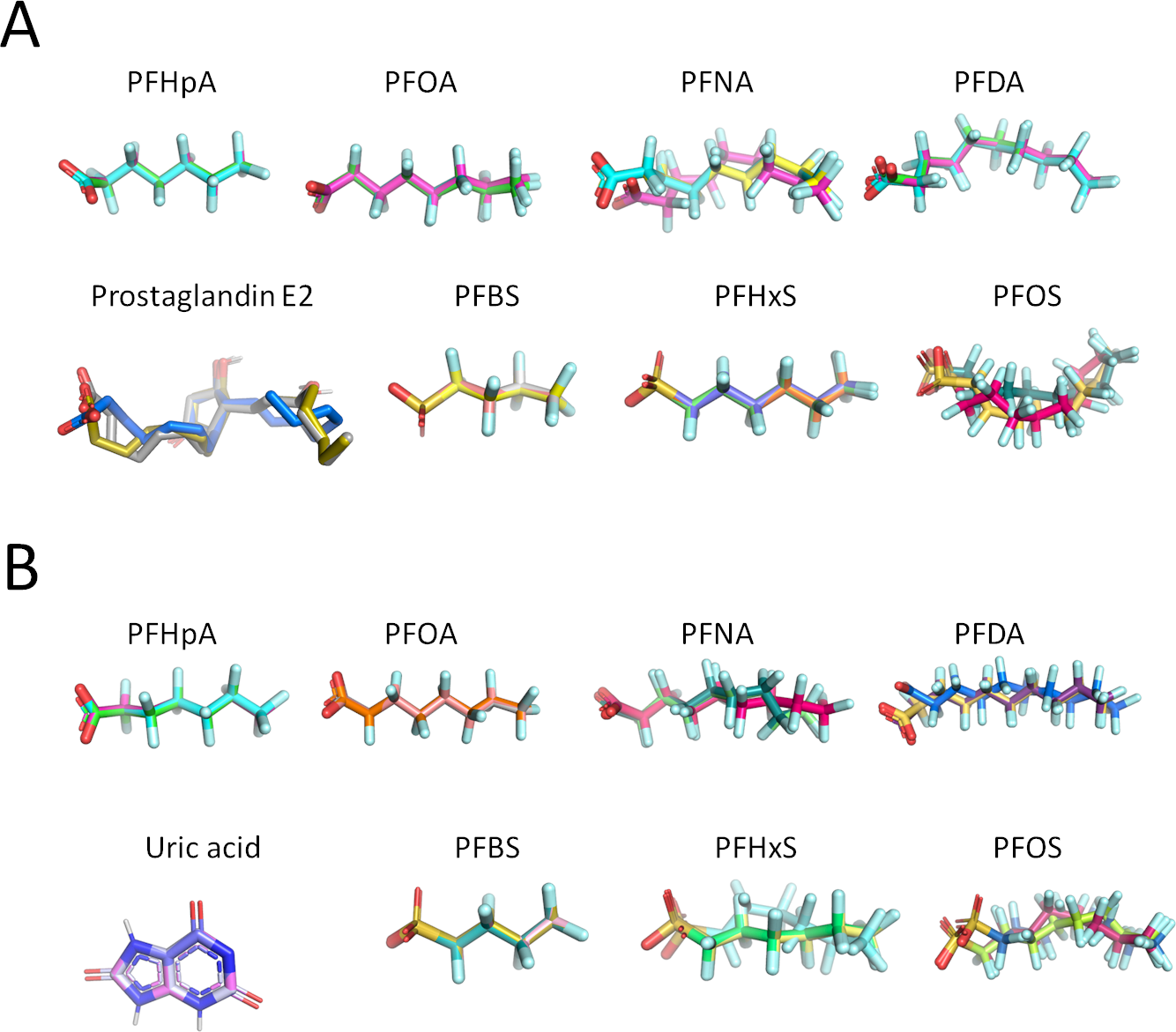


#### Supplementary Figure 2. Superimposition of the three docking poses calculated for each molecule under analysis within OAT4 (A) or URAT1 (B).
